# Co-Localized Dermoscopy and LC-OCT for AI-Assisted Margin Assessment of Basal Cell Carcinoma: Development of a “BCC-One-Stop-Shop” Workflow

**DOI:** 10.3390/diagnostics16050750

**Published:** 2026-03-03

**Authors:** Marco Mozaffari, Clara Tavernier, Jonas Ogien, Pierre Godet, Kristina Fünfer, Hanna Wirsching, Maximilian Deußing, Elke Sattler, Julia Welzel, Sandra Schuh

**Affiliations:** 1 Department of Dermatology and Allergology, University Hospital, 86179 Augsburg, Germany; 2 Damae Medical, 75013 Paris, France; 3 Department of Dermatology and Allergy, Ludwig Maximilian University, 80337 Munich, Germany; 4 Department of Dermatology, Allergology and Laser Medicine, Munich Municipal Hospital, 80337 Munich, Germany

**Keywords:** basal cell carcinoma, non-melanoma skin cancer, line-field confocal optical coherence tomography, micrographic surgery, skin imaging, non-invasive diagnostics in dermatology, one-stop-shop

## Abstract

**Background/Objectives**: The surgical treatment of basal cell carcinoma (BCC) remains challenging due to the time-consuming, expensive and invasive nature of Mohs micrographic surgery. The objective is to develop a standardized protocol for managing diagnosis, surgery, and margin control within a single patient visit. **Methods**: Several protocols were tested to establish a “BCC-One-Stop-Shop”, combining in vivo and ex vivo margin mapping of BCC, pre- and postoperatively using Line-field confocal optical coherence tomography (LC-OCT). We introduce an algorithm enabling real-time localization of LC-OCT acquisitions on a previously acquired dermoscopy image. Additionally, an artificial intelligence model provides a BCC probability score based on LC-OCT images. Together, the co-localization algorithm and AI BCC model generate a color-coded visualization of the tumor within the dermoscopy image, allowing precise pre-operative in vivo margin assessment. **Results**: We found our protocol, the implementation of the co-localization tool and the AI model, to be quick to apply, easy to learn and helpful regarding the initial determination of BCC tumor margins. Patients responded positively to the recognizable visualization of the disease. **Conclusions**: Pre- and postoperative margin mapping using LC-OCT imaging appears to be effective and feasible and could reduce time, costs, resources, excision sizes and patient burden by sparing additional excision steps in micrographic surgery. The integration of real-time co-localization and the AI-calculated probability score represent meaningful and practical enhancements for routine clinical use. To further evaluate the efficacy and safety of the BCC-One-Stop-Shop-Method and the newly introduced device features, larger-scale studies are warranted and are currently being conducted.

## 1. Introduction

The perioperative margin mapping of basal cell carcinoma (BCC) has been a major research focus in dermatology over the last decade [1,2,3]. Given the high and steadily rising incidence of BCC, this line of research is becoming increasingly relevant to patient care [4,5]. The development of BCC is closely associated with cumulative lifelong UV exposure, and owed to their increased sun exposure, the head and neck regions are most frequently affected [6]. Because of local tissue invasion and destruction, the extension of BCC can sometimes be subclinically more severe, making preoperative margin assessment challenging and increasing the risk of inadequate excision [7]. This in turn may necessitate additional surgeries, resulting in higher costs for the healthcare system as well as a greater burden for patients [8]. Currently, surgical removal remains the standard of care, offering the highest cure rates compared with other treatment options.

However, standard surgical excision has relevant limitations that should be acknowledged. Current guidelines of the European Association of Dermato-Oncology (EADO, 2023) recommend safety margins due to the existing risk factors, dividing BCC into EADO stages I–IV [9]. For a large proportion of BCC, particularly those located on the central face, the nose or the ear, aggressive histological subtypes, or recurrent tumors, are assigned as stage II, a wide safety margin of >5 mm is recommended [9]. As a consequence, extensive excisions are frequently required in cosmetically and functionally sensitive areas. Despite these wide margins, recurrence rates after standard excision remain clinically relevant, indicating that conventional surgery for specific patients and lesions may be both insufficiently accurate at the microscopic level while simultaneously sacrificing excessive healthy tissue [10]. This highlights an inherent limitation of two-dimensional histopathological margin assessment and underlines the need for optimized surgical and histological protocols that may benefit not only micrographic surgery but also standard excision techniques [9]. Mohs micrographic surgery (MMS), in particular, was introduced to minimize the extent of surgical excision while maintaining excellent cure rates [11]. However, the very principle of MMS—preserving as much healthy tissue as possible—introduces a key disadvantage: multiple surgical stages are often required to ensure complete tumor clearance [12,13]. Each stage relies on histopathological processing and the expertise of specially trained professionals, typically within a clinical setting [14]. Consequently, this approach can be resource-intensive and impose considerable psychological stress for the patient [8,15]. Importantly, the protocol described in this study is not limited to Mohs or slow Mohs surgery, but may also be applicable to standard surgical excision, potentially improving margin control and tissue preservation independent of the surgical technique used.

With the advent of non-invasive imaging devices such as optical coherence tomography (OCT), reflectance confocal microscopy (RCM), and line-field confocal optical coherence tomography (LC-OCT), non-invasive imaging of skin lesions has become possible. OCT and, more recently, LC-OCT are now routinely used in clinical practice for the diagnosis of BCC, whereas RCM is more commonly applied to melanocytic lesions [16,17,18,19]. The diagnostic sensitivity and specificity of OCT and LC-OCT for BCC have been well documented in published studies [17,18,20].

The precise visualization of BCCs histological features within the noninvasive imaging by LC-OCT raises the question of whether these characteristics can also be assessed at the suspected lateral tumor margins, in addition to the central lesion [21,22,23,24]. If BCC margins could be accurately identified prior to the first step of MMS, excision could be performed more efficiently, resulting in shorter patient hospitalization and optimized use of staff and operating room resources. Several non-invasive imaging modalities have already been investigated, and recent studies have demonstrated that margin examination with OCT and RCM is more accurate than clinical or dermoscopic assessment alone [25,26,27,28].

Recent advances in LC-OCT have continued to expand its clinical utility for BCC diagnosis and characterization. Boussingault et al. (2025) demonstrated systematic correlation between LC-OCT imaging and histopathology in BCC cases, supporting the technique’s high resolution and diagnostic validity across different histologic subtypes [29]. Similar robustness was observed in large data sets [29]. In a large retrospective study, Mtimet et al. (2024) reported that LC-OCT significantly improved diagnostic performance compared with clinical and dermoscopic evaluation alone, emphasizing its potential to refine lesion classification in routine practice [22]. More recently, Orte Cano et al. (2026) provided prospective evidence for the diagnostic accuracy of LC-OCT in differentiating BCCs at the bedside, further validating its role outside strictly controlled research settings [30]. Complementary retrospective data suggest that artificial intelligence (AI)-assisted LC-OCT interpretation may further enhance diagnostic accuracy and consistency, potentially facilitating broader clinical adoption [31]. However, variability in interobserver agreement for key LC-OCT image markers has also been reported, highlighting a need for standardized workflows and reproducibility across centers [32]. Taken together, these studies underscore the promising clinical performance of LC-OCT but also reveal that robust, step-by-step workflows integrating wide-field dermoscopic co-localization and reproducible acquisition strategies remain underdeveloped. Such methodological gaps limit the systematic application of LC-OCT across different clinical settings and justify the need for the present study, which focuses on establishing a standardized and reproducible LC-OCT-based “One-Stop-Shop” workflow for BCC.

From a practical and procedural perspective, the translation of LC-OCT from diagnostic studies into perioperative margin assessment remains insufficiently defined. While individual technical capabilities of LC-OCT have been demonstrated, there is currently no consensus on how margin assessment should be operationalized in daily clinical routines, including lesion preparation, acquisition order, orientation, and documentation of examined margin segments. In addition, the lack of systematic surface co-localization limits the reproducibility of LC-OCT findings and complicates comparison between examinations, operators, and centers. Finally, structured visualization concepts that support clinicians during margin assessment—such as intuitive, real-time highlighting of suspicious areas—are still rarely incorporated into existing LC-OCT applications.

The present study addresses these limitations by introducing a structured and reproducible workflow for LC-OCT–based perioperative margin assessment of basal cell carcinoma. By combining wide-field dermoscopic co-localization for precise acquisition-path documentation with AI-supported visualization of BCC probability, this approach emphasizes workflow standardization, reproducibility, and clinical feasibility. The proposed “BCC-One-Stop-Shop” concept is conceived as a structured procedural framework that enables consistent in vivo margin assessment within a single patient visit and provides a foundation for future interventional applications.

In this paper, we introduce a method enabling in vivo margin mapping of BCC based on LC-OCT imaging co-localized with wide-field dermoscopy. Using this approach, clinically determined lateral margins can be evaluated in vivo prior to surgical excision, thereby providing a structured foundation for standardized perioperative margin assessment. An AI algorithm generates a BCC score based on LC-OCT imaging. This score is displayed on the wide-field dermoscopy image as a color code, allowing rapid identification of zones that still present BCC imaging features. The technical details of the LC-OCT system and the video-dermoscopy system, the co-localization algorithm and the AI-based visualization strategy are described. The AI algorithm and the method for mapping a color-coded BCC score on the dermoscopy image are subsequently introduced. Based on this method, the objective of this study is to establish and preliminarily evaluate a standard operating procedure (SOP) for the “BCC-One-Stop-Shop-Method” workflow, focusing on in vivo LC-OCT based margin assessment within a single patient visit. The protocol is applied in an observational manner, serving as a prerequisite for future interventional applications. A standardized approach is needed for future research, larger multicenter trials, and as a practical guidance for routine clinical practice. As post-operative ex vivo margin assessment can also be useful, an ex vivo SOP was designed, based on a previously introduced ex vivo prototype compatible with the same LC-OCT system used for in vivo margin determination. Applications of this SOP are presented in this paper for some relevant clinical cases.

## 2. Materials and Methods

### 2.1. LC-OCT Device

We used the CE-certified LC-OCT device deepLive™ (DAMAE Medical, Paris, France), which enables non-invasive, real-time, high-resolution grayscale imaging of the skin. The technical details of this system have been described previously [33]. Briefly, the device is based on a time-domain OCT and uses a supercontinuum laser for line illumination and detection, combined with microscope objectives (20×, NA 0.5) to achieve high resolution imaging and (line-) confocal filtering. The laser line is split into two paths: one directed toward the skin and the other toward a reference surface. Light is backscattered from subsurface skin structures and reflected from the reference surface. The light from both paths is then recombined and imaged onto a line-scan camera (single pixel array), generating an interference pattern. The intensity of this interference depends on the backscattering intensity of the skin structures at a given depth. Using a demodulation algorithm, the interference signal is processed to generate a one-dimensional (line) intensity image of the skin at a specific depth [34]. To obtain a full two-dimensional (2D) image, the line is scanned either vertically (from the skin surface to deeper layers) by vertically scanning the interferometer using a piezoelectric actuator, or horizontally (within a plane parallel to the skin surface at a fixed depth) by scanning the laser beam using a galvanometer. Line images acquired during these scans are concatenated to form 2D images. 3D images are obtained by stacking horizontal images acquired while the interferometer is vertically scanned with a step size of 1 µm between successive images.

LC-OCT thus enables the acquisition of vertical images (B-scans, 1.2 mm × 0.4 mm) and horizontal images (C-scans, 1.2 mm × 0.5 mm) in real time (8 frames per second), as well as 3D volumes (1.2 mm × 0.5 mm, up to 0.5 mm in depth), which are acquired in approximately 25 s reaching the maximum depth. Videos of vertical or horizontal image sequences can be recorded. The spatial resolution achieved by LC-OCT is 1.3 µm (laterally) × 1.1 µm (axially) [34].

The device also includes a secondary optical path that enables simultaneous acquisition of color images of the skin surface during LC-OCT imaging, at the same frame rate (8 frames per second) [35]. This secondary path incorporates white-light LEDs at the distal end of the probe. The LED light is diffusely reflected by the skin surface and collected by the microscope objective used for LC-OCT imaging. The light then passes through the galvanometer scanner positioned upstream of the objective, with a beamsplitter mounted on the galvanometer, and is imaged onto a 2D color camera by an optical system. A 600 nm low-pass spectral filter (Edmund Optics Inc., Barrington, NJ, USA) is included to attenuate the supercontinuum laser in this optical path. A micro-objective with adjustable focus is mounted on the color camera, allowing the surface image to remain in focus even during axial scanning of the interferometer. The field of view (FOV) of the surface images is a circular, with a diameter of 2.6 mm. The spatial resolution of the images is approximately 6 µm, comparable to that of a 10-fold magnification dermoscope. A marker is overlaid on the surface image to indicate the location of the LC-OCT acquisition: a line is displayed for vertical LC-OCT images, while a rectangle is displayed for horizontal images or 3D volumes.

The complete optical system, including the LC-OCT path and secondary optical path, is integrated into a lightweight handheld probe (860 g) with a compact tip, allowing easy access to almost all regions of the body.

### 2.2. Co-Localized Dermoscopy

#### 2.2.1. Video-Dermoscope

**Figure 1:**
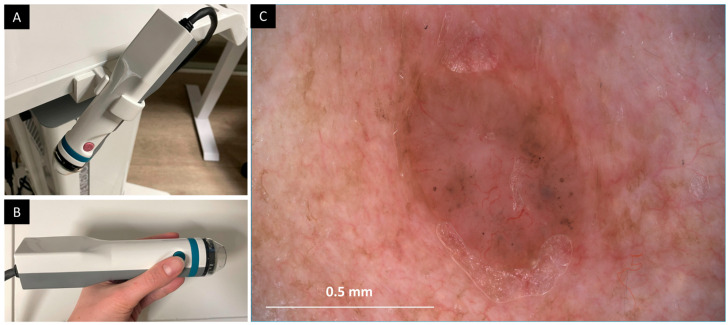
Video-dermoscope used for co-localized imaging. (**A**,**B**) Photographs of the deepLive^TM^ video-dermoscope. (**C**) Example of a polarized contact dermoscopic image of a basal cell carcinoma with the device (paraffin oil used as immersion medium).

To better guide LC-OCT evaluation of large skin areas, an additional dermoscopic image is required that is substantially larger than the previously introduced surface image. Image acquisition guidance is achieved by co-localizing the LC-OCT acquisition within a video-dermoscopic image acquired prior to the LC-OCT examination. The video-dermoscope used is a CE-certified deepLive™ accessory (DAMAE Medical, Paris, France). Figure 1 shows pictures of the video-dermoscope, along with an example image obtained using this device.

The deepLive^TM^ video-dermoscope is based on contact dermoscopy. Its tip is similar in size to that of the deepLive^TM^ LC-OCT probe. The skin is illuminated by white LEDs integrated into the tip, allowing either unpolarized or linearly polarized illumination. An optical system images the diffusely reflected light from the skin surface onto a color camera equipped with a 20-megapixel, 1-inch sensor. Buttons on the video-dermoscope allow the user to capture images and to switch between unpolarized and polarized dermoscopy.

The resulting images cover a FOV of 13.3 × 8.9 mm^2^ with a spatial resolution of ~3.5 µm, comparable to the FOV and resolution of a 25-fold magnification dermoscope.

Images are displayed in real time (8 frames per second), in the same software used for LC-OCT acquisition.

Table 1 summarizes the key imaging parameters of both the deepLive^TM^ video-dermoscope and the deepLive^TM^ LC-OCT device, including both the LC-OCT imaging path and the secondary optical path (color surface imaging).

For some lesions, the FOV of the video-dermoscope may be insufficient to cover the entire lesion. To extend the FOV, a mosaicking mode is implemented in the software, allowing the user to acquire a series of images that are automatically combined into a dermoscopic mosaic covering the full extent of the lesion. When mosaicking mode is activated and the first image has been captured, a marker shows the position of the current video-dermoscope FOV relative to the initial image in real time, enabling the user to extend the coverage without missing any regions. Each subsequentially acquired image is automatically stitched to the previously recombined images. The user may stop the acquisition once the full extent of the lesion and its margins has been reached. Up to 40 images can be stitched together to form a mosaic.

#### 2.2.2. Co-Localization

To localize the LC-OCT acquisition within the video-dermoscopic image, a co-localization algorithm was developed to register the surface image (acquired via a secondary optical path of the LC-OCT probe) to the video-dermoscope image, based on a combination of a Scale-Invariant Feature Transform (SIFT) algorithm and a Random Sample Consensus (RANSAC) algorithm [36]. Because the LC-OCT acquisition is spatially referenced within the surface image, localizing the surface image within the video-dermoscope image also allows localization of the LC-OCT acquisition itself.

The algorithm begins with an initialization step in which the video-dermoscope image is processed using the SIFT algorithm [37]. SIFT is applied to detect key points and compute corresponding descriptors, which are subsequently used for matching with descriptors extracted from the surface image.

For each frame in the real-time surface image stream, SIFT descriptors are computed in the same manner and matched to those of the video-dermoscopic image. Each descriptor from the surface image is associated with its closest match in the video-dermoscopic image based on the minimum Euclidean distance between descriptor vectors, forming descriptor pairs. A RANSAC algorithm is then applied to estimate a unique geometric transformation (comprising translation and rotation) that best aligns the matched pairs [38]. Descriptor pairs that do not conform to this transformation are discarded. The number of remaining inlier pairs is an indicator of the confidence of the localization result. We have empirically identified that when the number of remaining inliers is above 6, the result can be regarded reliable. Therefore, a threshold of 6 is applied on the remaining inliers, below which the result is considered unreliable and is automatically rejected.

To improve robustness, a secondary algorithm based on a similar SIFT approach is employed to estimate inter-frame motion between successive images in the real-time surface images stream. In cases where a reliable localization has been obtained but the confidence score subsequentially falls below the threshold, inter-frame motion estimation is used to extrapolate the position of the surface image, thereby maintaining effective co-localization. This inter-frame tracking also produces a confidence score; if this score drops below a predefined threshold, temporal tracking is reset. Such situations typically occur when contact between the probe and the skin is lost or when the probe is moved too rapidly.

When the surface image is successfully co-localized within the video-dermoscopic image, an overlay marker is displayed to the user on the video-dermoscopic image. This marker indicates the position of the surface image, the position of the LC-OCT acquisition, and an arrow showing the orientation of the probe relative to the video-dermoscopic image.

### 2.3. AI-Based In Vivo BCC Mapping

In addition to localizing the LC-OCT acquisition within the video-dermoscopic image, a method is proposed to directly display on the video-dermoscopic image a score representing the probability of basal cell carcinoma (BCC) presence. This score is computed using an artificial intelligence (AI) model, CE-certified as an extension of the deepLive™ system.

The model [31] generates a BCC score (expressed as a probability in %) for each vertical LC-OCT image, within the real-time imaging stream, thereby providing immediate feedback to the user. In addition to the numerical score, the model generates a heatmap that is overlaid on the LC-OCT images [31]. The heatmap is obtained from applying the AI model to a number of sub-regions (patches) in the LC-OCT image, resulting in a BCC score for each patch. A threshold is then applied to keep only the patches above a certain score, highlighting image regions suspected of containing BCC-related features. The retained patches are colored based on the global BCC score of the image, ranging from blue (score = 0%) to yellow (score = 100%) [31]. For example, in an image with a global BCC score of 78%, patches exceeding the predefined threshold are displayed in yellowish tones, indicating regions with high suspicion of BCC-related features, whereas patches below the threshold are suppressed.

The BCC model was trained on a large dataset comprising 685,000 LC-OCT images acquired from 1154 lesions in 1047 patients. Its performance was further evaluated and validated through a reader study involving 43 dermatologists, who independently reviewed the same test set of 200 equivocal BCC cases. The study demonstrated that LC-OCT significantly improves dermatologists’ diagnostic performance for BCC detection, with increases of +25.8 percentage points in sensitivity and +16.8 percentage points in specificity compared with clinical and dermoscopic examination. Further details about training set, test set, and model performances are available in a specific study conducted by Fischman et al. [31].

To display the AI-generated BCC score on the video-dermoscopic image, the color of the marker indicating the position of the LC-OCT acquisition is modulated according to the BCC score. As with the heatmap, the marker color ranges from blue (score = 0%) to yellow (score = 100%). This visual encoding allows rapid identification of skin regions at risk of BCC with minimal need for detailed analysis of the LC-OCT image.

**Figure 2:**
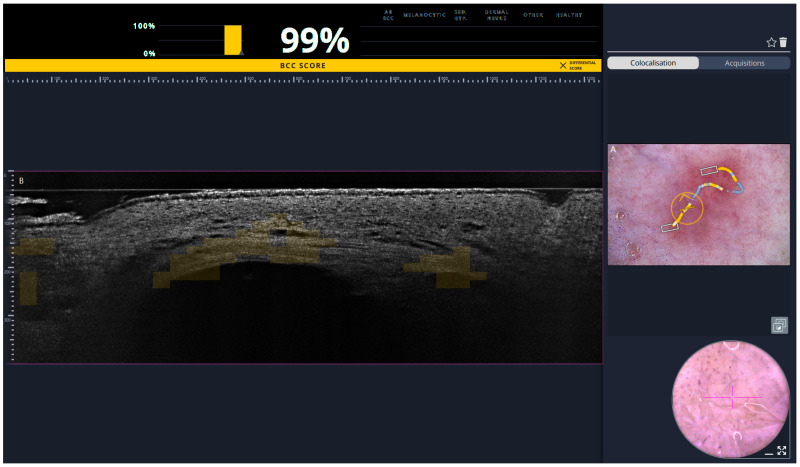
Co-localized dermoscopy and LC-OCT imaging. (**A**) Contact dermoscopic image acquired with the deepLive^TM^ dermoscope. (**B**) Vertical Line-field confocal optical coherence tomography (LC-OCT) video sequence acquired for the diagnosis of a basal cell carcinoma (BCC) (histological tumor diameter: 1 cm). The lesion size corresponds to the measurement reported in the histopathological examination after excision. The color of the marker on the dermoscopic image indicates the position of the LC-OCT acquisition and is modulated according to the AI-generated BCC score (ranging from blue = 0%, to yellow = 100%). The circle demonstrates the exact location of the LC-OCT measurement in the dermoscopic overview in real-time. The squares represent areas where 3D stacks were taken. The example is intended to demonstrate the technical robustness of the co-localization algorithm and AI-supported mapping system, independent of tumor size.

In particular, when the user records a video of LC-OCT vertical images while moving the probe across skin, the trajectory of the probe on the skin surface—reconstructed using the co-localization algorithm—is recorded and displayed. The color along this path reflects the BCC score computed at each probe position during the acquisition. An example illustrating coverage of a BCC lesion using this approach is shown in Figure 2.

## 3. Results

We present here our SOP for in and ex vivo margin assessment.

### 3.1. In Vivo Pre-Operative Margin Assessment

We developed a structured study protocol and refined it to ensure seamless integration into daily routines, enabling reliable perioperative in vivo deepLive™ (DAMAE Medical, Paris, France) LC-OCT-based margin assessment within routine clinical workflows. The protocol was designed to apply and assess feasibility of this approach in an observational manner, without preoperative modification of surgical margins. The LC-OCT is combined with a contact video-dermoscope accessory and an AI-based algorithm for BCC detection. This approach allows a fast margin assessment with a promising histological correlation. The measurements took an average of 10 and 20 min, excluding the drying time of the color markings. The total duration varied depending on lesion size, skin texture, and anatomical location.

#### 3.1.1. Margin Drawing and Dermoscopic Mapping

##### In Vivo Workflow (Step-by-Step)

Step 1: Skin preparation and margin marking

**Figure 3:**
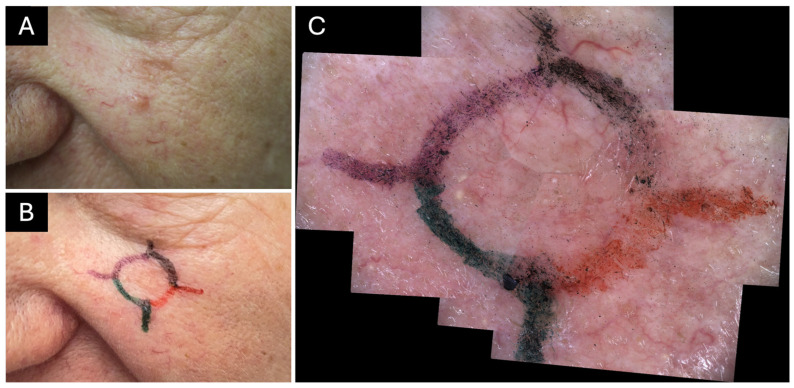
Clinical and dermoscopic margin delineation prior to Line-field confocal optical coherence tomography (LC-OCT) imaging. (**A**) Clinical image of a basal cell carcinoma (BCC) (histological tumor diameter: 0.7 cm) on the left cheek without margin delineation. The tumor diameter corresponds to the measurement reported in the histopathological examination after surgical excision. (**B**) Clinical image with dermoscopically defined margins of the same BCC with a 2–3 mm safety distance. Each quadrant is marked in a different color (black. 12–3 o’clock, red: 3–6 o’clock, green: 6–9 o’clock, purple: 9–12 o’clock) for standardized margin assessment. (**C**) Multiple contact dermoscopic images acquired with the deepLive^TM^ video-dermoscope and automatically combined into a dermoscopic mosaic, allowing visualization of the entire lesion and its margins within a single image.

Prior to LC-OCT imaging, the skin is cleaned with an alcoholic disinfectant. The lesion is then examined clinically and dermoscopically by a dermatologist using a standard dermoscope. Presumed BCC margins are marked around the lesion with a safety distance of 2–3 mm using tattoo pens (BodyMark BIC, Clichy, France). For margin delineation, a recommended surgical skin-marking pen was used, as its pigment does not introduce artifacts or signal attenuation in LC-OCT imaging. For subsequent correlation and standardization, each quadrant of the lesion (12–3, 3–6, 6–9, 9–12 o’clock) is assigned to a specific color to enable standardized orientation throughout the workflow (Figure 3).

Step 2: Drying of the markings

The markings are allowed to dry for 5–10 min, depending on the individual skin texture (this preparation time is not included in the average LC-OCT measurement duration of 10–20 min) to ensure color stability and avoid smudging during subsequent steps.

Step 3: Paraffin oil application (pre-processing)

A thin layer of paraffin oil is applied to the marked area to optimize optical coupling for dermoscopy and LC-OCT imaging.

Step 4: Wide-field dermoscopic documentation

A contact dermoscopic image is taken using the video-demoscope of the deepLive™ system. If the lesion and its margins extend beyond the video-dermoscope’s FOV, multiple overlapping images are captured and automatically combined into a dermoscopic mosaic; sufficient overlap between consecutive images is ensured for accurate reconstruction and indicated to the investigator by frames created in the software (Figure 3).

#### 3.1.2. LC-OCT Imaging

Step 5: LC-OCT system start

The LC-OCT system is switched on and used directly for imaging (no calibration step required). Once the video-dermoscopic image or mosaic has been acquired, the user switches to LC-OCT imaging for in-depth, cellular-resolution evaluation of the lesion margins. The software automatically switches between video-dermoscope and LC-OCT imaging when the user clicks the capture button on either probe. If necessary, additional paraffin oil can be reapplied on the margins to prevent bubbles.

Step 6: Sequential LC-OCT margin imaging (quadrant-based)

**Figure 4:**
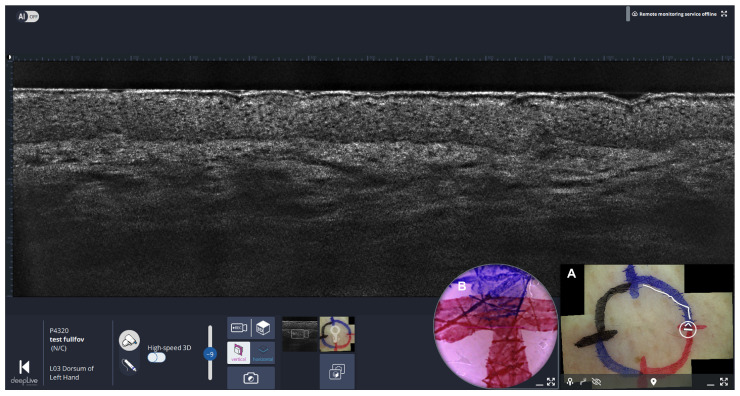
Line-field confocal optical coherence tomography (LC-OCT) imaging workflow guided by co-localized dermoscopy. Screenshot of the deepLive^TM^ software (version 2.0.1) in live mode. The dermoscopic mosaic picture (lower right (**A**)) displays a circular white marker indicating the real-time position of the LC-OCT probe. Next to the dermoscopic mosaic, a real-time camera view (**B**) shows the patient’s skin and allows examination of the entire lesion along the previously marked margins. The white line indicates areas where LC-OCT video sequences were previously recorded.

LC-OCT video sequences are recorded sequentially along the previously marked lateral margins in each quadrant, following the colored lines in a continuous manner to avoid gaps (Figure 4). During acquisition, co-localization with the dermoscopic overview is used to track the probe position and ensure complete margin coverage.

Step 7: Quality checks during acquisition

To ensure reliable co-localization and optimal image quality, air bubbles and excess oil must be minimized. If co-localization deteriorates because of insufficient skin texture or altered margin markings, the marking and imaging procedures should be repeated as required.

Step 8: Tumor center imaging after margin mapping

After completion of margin assessment, additional LC-OCT images/videos and a 3D stack of the tumor center are acquired to assess tumor penetration depth and subtype.

No preoperative adjustments of the surgical margins were performed based on the LC-OCT findings, as this study was conducted as an observational feasibility study in accordance with the requirements of the local ethics committee.

#### 3.1.3. Final LC-OCT Review

**Figure 5:**
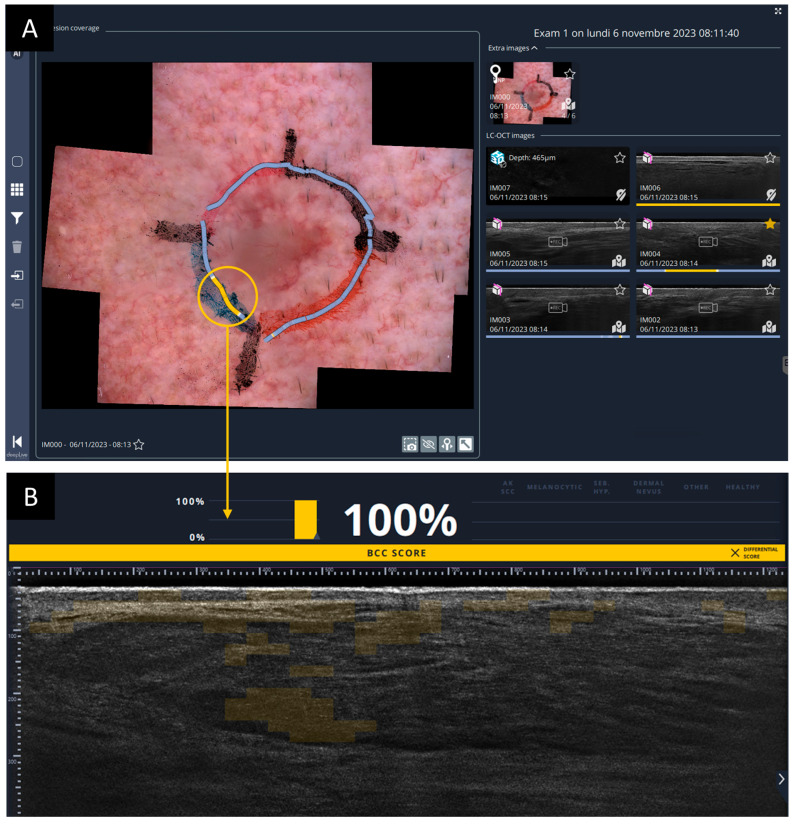
Artificial intelligence (AI)-assisted Line-field confocal optical coherence tomography (LC-OCT) margin assessment. (**A**) End of the examination showing complete lateral margin coverage of a basal cell carcinoma (BCC) with LC-OCT. The color-coded LC-OCT acquisition path overlaid on the dermoscopic image allows easy tracking of the examined areas and indicates the AI-generated BCC probability score (blue = low probability, yellow = high probability). (**B**) Corresponding vertical LC-OCT image with a positive AI assessment, showing a superficial BCC lobule highlighted by the AI-generated heatmap.

After image acquisition, all LC-OCT images should be reviewed by the user to confirm the presence or absence of residual BCC at the margins. AI supports this process by providing an initial assessment. The AI output is visualized on the dermoscopic image as a color-coded representation of the LC-OCT acquisition path along the margins. An example of complete margin coverage of a BCC lesion using this approach is shown in Figure 5A. In this case, the path is predominantly blue, indicating no BCC detected by the AI algorithm, with the exception of a localized yellow segment in the southwest quadrant, suggesting possible BCC involvement. These yellow areas are of particular interest and should be prioritized during image review. To confirm the actual presence of BCC, the corresponding LC-OCT images should be carefully examined, with particular attention to the highlighted heatmap regions, to verify whether true BCC lobules are identifiable, as illustrated in Figure 5B.

Once the in vivo preoperative assessment is completed, the patient is transferred directly to the operating room, where the lesion is excised according to the margins previously delineated during the LC-OCT process using the slow Mohs micrographic surgery technique, which is routinely applied in Germany.

### 3.2. Ex Vivo Margin Assessment

In this study, we also explored an ex vivo standard operating procedure (SOP) using an LC-OCT ex vivo prototype. After resection, the tissue was placed in saline solution to preserve its integrity, optical properties, and chemical composition for optimal imaging.

Ex vivo LC-OCT imaging was performed using a prototype previously described [39]. This system uses the same probe as that employed for in vivo imaging; however, the probe tip is removed and replaced with a component that allows the probe to be mounted vertically on a platform equipped with a motorized XY microscope stage and compatible with a custom sample holder.

Ex vivo LC-OCT workflow (step-by-step):

Step 1: Specimen orientation and handling

Immediately after excision using slow Mohs micrographic surgery, the specimen is oriented according to the surgical markings and transferred to the ex vivo LC-OCT setup. Care needs to be taken to preserve the orientation of the lateral margins. The excised tissue is placed in the sample holder beneath a transparent glass window (3 cm in diameter).

Step 2: Preparation of the ex vivo imaging interface

A thin layer of paraffin oil is applied as a contact medium between the tissue and the glass window to ensure optimal coupling and to avoid air interfaces. The sample is secured using a magnetic compression plate with customized foam inserts. Silicone oil is applied between the probe tip and the glass window as an immersion medium.

Step 3: Placement of the specimen

The excised tissue is positioned on the ex vivo sample holder with the lateral margin facing the probe window. Gentle and uniform contact is necessary to avoid tissue deformation.

Step 4: System start and overview positioning

The LC-OCT system is switched on and used directly for imaging. Once mounted, the specimen can be moved freely beneath the LC-OCT probe using the motorized stage via joystick-controlled motors. The software is adapted to control the stage and provides several imaging modes, including a “scout mode” for generating a mosaic image of the surface to obtain an overview of the full aperture of the sample holder, as well as LC-OCT mosaic modes that allow multiple LC-OCT images to be stitched together to increase the FOV. 3D volumes of up to 5 × 5 × 0.5 mm^3^ can be obtained. When an LC-OCT mosaic is generated, a corresponding mosaic of the associated surface images is also produced simultaneously.

Step 5: Sequential ex vivo margin imaging

**Figure 6:**
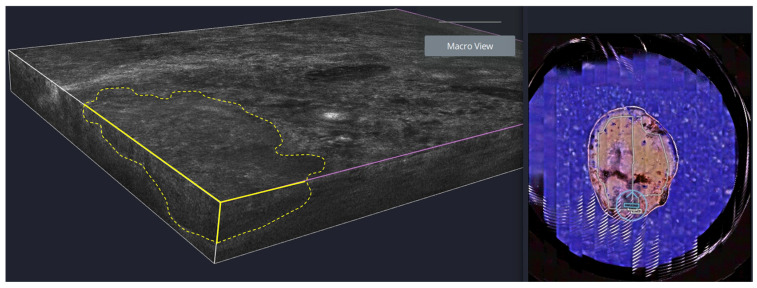
Ex vivo Line-field confocal optical coherence tomography (LC-OCT) assessment of surgical margins of a basal cell carcinoma (BCC). (**Left**): Ex vivo 3D LC-OCT stack of a BCC-suspicious lateral margin, with residual tumor tissue outlined in yellow. (**Right**): Scout mode image providing an overview of the excised specimen. The blue marker indicates the position within the specimen from which the 3D LC-OCT stack was obtained.

LC-OCT video sequences and 3D stacks were acquired sequentially along the entire lateral margin of the specimen for each quadrant. Imaging is performed in a continuous manner to ensure complete margin coverage without gaps. Suspicious regions are further examined using high-resolution 2D and 3D mosaics. The “scout mode” is also used to confirm that the excised specimen corresponds to the margins planned during the in vivo assessment (Figure 6). For the evaluation of the deep margin (basis), the specimen is flipped and then imaged upside-down.

Step 6: Image quality control during acquisition

During imaging, air bubbles, excessive oil, or loss of optical contact need to be avoided. If image quality is insufficient, the specimen position or oil layer has to be adjusted before continuing acquisition.

Step 7: Correlation with histopathology

After completion of ex vivo LC-OCT imaging, the tissue is returned to the pathology laboratory for conventional formalin fixation and histological analysis performed using either the Tübingen cake (“Tübinger Torte”) technique or the Munich Method, in accordance with slow Mohs micrographic surgery protocols. This enables direct correlation between LC-OCT findings and histological margin status. The complete in and ex vivo LC-OCT workflow does not induce tissue damage or alter tissue morphology, allowing subsequent analysis using standard histopathological methods.

### 3.3. Some Examples

The in vivo and ex vivo SOP was applied to 50 lesions between 11 October 2023 and 19 November 2024. All lesions were excised using the slow Mohs micrographic surgery technique, which is routinely performed at our center. The SOP method was rapid (10–20 min) and easy to integrate into daily clinical practice. A positive patient reception was observed, particularly due to the imaging-based information used to explain the planned surgery. Although the performance results of this study will be reported in a separate publication, two representative cases illustrating different clinical scenarios are presented here.

**Figure 7:**
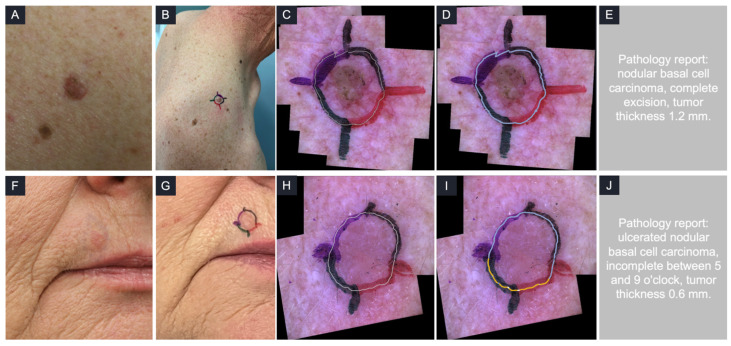
Representative clinical cases illustrating LC-OCT margin assessment. Case 1: (**A**): Basal cell carcinoma (BCC) on the right shoulder of a 59-year-old man. (**B**) Clinical and dermoscopic margin delineation of the same BCC with a safety distance of 2–3 mm. (**C**) Dermoscopic mosaic showing the LC-OCT acquisition path (white line). (**D**) AI-based color-coded LC-OCT acquisition path indicating negative lateral margins. (**E**) Histopathological assessment following slow Mohs micrographic surgery confirming complete (R0) excision. Case 2: (**F**) BCC located above the right upper lip of a 65-year-old woman. (**G**): Clinical and dermoscopic margin delineation of the marked BCC with a safety distance of 2–3 mm. (**H**) Dermoscopic mosaic with LC-OCT acquisition path (white line). (**I**) AI-based color-coded LC-OCT acquisition path indicating positive margins between 5 and 9 o’clock. (**J**) Histopathological assessment following slow Mohs micrographic surgery confirming incomplete (R1) excision corresponding to the positive LC-OCT findings.

The first case, illustrated in Figure 7A, involved a 5 mm lesion located on the right shoulder in a 59-year-old man. Following in vivo LC-OCT mapping, the surgical margins were classified as negative by the AI-assisted LC-OCT system, a finding that was confirmed by the dermatologist. The patient then proceeded directly to the first stage of slow Mohs surgery. The excised tissue was imaged ex vivo using the LC-OCT prototype, which again confirmed the absence of tumor at the lesion margins. Subsequent histological examination demonstrated a complete (R0) excision. In this case, LC-OCT accurately predicted complete tumor removal.

The second case, illustrated in Figure 7B, involved a 4 mm lesion located on the right side above the upper lip in a 65-year-old woman. In vivo LC-OCT mapping identified two quadrants as positive on AI-assisted analysis, between 5 and 9 o’clock. The presence of BCC lobules on the LC-OCT images was confirmed by the dermatologist. The patient then underwent the first stage of slow Mohs surgery. Ex vivo LC-OCT imaging of the excised specimen confirmed tumor involvement at the corresponding margin. Histological analysis subsequently demonstrated an incomplete (R1) excision between 5 and 9 o’clock. In this case, LC-OCT correctly anticipated that the first stage of slow Mohs surgery would be incomplete.

## 4. Discussion

Mohs micrographic surgery (MMS), the current gold standard for basal cell carcinoma (BCC) treatment, achieves significantly lower recurrence rates (approximately 1–3%) compared with standard surgical excision, particularly for high-risk and facial tumors [10,13]. Such outcomes may necessitate multiple surgical steps, increasing staff workload costs, prolonging hospital stays, and causing significant psychological stress for patients [11]. Given the steadily rising incidence of BCC, there is a clear need for a more individualized treatment approach that is skin-, time- and resource-saving.

In most parts of the world, dermoscopy is the primary diagnostic tool used for BCC diagnosis and preoperative margin assessment [40]. Considerable research has focused on combining Mohs micrographic surgery with preoperative imaging tools, such as conventional dermoscopy alone, optical coherence tomography, confocal laser microscopy as well as high-resolution ultrasound devices [23,25,26,27]. The overall conclusion shows a good and important enhancement in daily practice and therapeutic pathways. However, no universally applicable or standardized protocol currently exists for preoperative BCC margin mapping using LC-OCT. Considering the excellent accuracy of LC-OCT in detecting BCC and its subtypes [17,30,31], we established a method that enables non-invasive margin characterization both in vivo (preoperatively) and ex vivo (postoperatively) using LC-OCT. This approach is consistent with recent studies demonstrating the applicability of LC-OCT for BCC assessment in anatomically challenging and special site locations, where precise margin delineation is particularly critical [21,41]. In the present study, margins identified as positive were not modified, as the protocol was applied in an observational manner to allow direct comparison between LC-OCT findings and histopathological results of slow Mohs surgery, as required by the initial ethics approval. Accordingly, the protocol presented in this study responds to the defined aim by establishing a structured and feasibility-tested workflow for LC-OCT-based margin assessment within a single patient visit, which constitutes a prerequisite for future interventional one-stop-shop applications. Given the limited number of illustrative cases presented in this manuscript, this evaluation should be understood as a preliminary feasibility assessment of workflow implementation rather than a formal statistical validation. Following further successful preliminary evaluation of the method, a second ethics approval has since been obtained, allowing LC-OCT-guided preoperative margin adjustment, and a prospective multicenter study evaluating this extended approach is currently being initiated. In such future interventional settings, if the presence of BCC is confirmed by LC-OCT examination at one or more margin sites, the dermatologist may adjust the surgical margins accordingly and re-image the newly defined margins using the same protocol until complete BCC margin negativity is achieved. Furthermore, this future study will provide a significant number of results for the co-localization algorithm presented here, from which performance indicators and potential sources of localization errors could be derived to assess its accuracy.

In line with existing research [31], we found this method to be quick, easy to implement in daily clinical practice, and well accepted by patients, who particularly appreciated the visual feedback regarding the otherwise often imperceptible tumor extent.

Against this background, comparison with recent LC-OCT–based studies help to further contextualize the present findings. When compared with recent LC-OCT based studies, the present work differs in both scope and focus. Previous investigations have primarily concentrated on diagnostic accuracy, histopathological correlation, or subtype differentiation of basal cell carcinoma using LC-OCT, thereby establishing the technical validity of the method [22,29,30]. More recent multicenter and AI-assisted studies have further demonstrated that LC-OCT, particularly when combined with artificial intelligence, can significantly improve diagnostic accuracy and consistency across clinicians with varying levels of expertise [31]. In addition, systematic reviews have positioned LC-OCT among the highest-performing non-invasive imaging techniques for BCC assessment when compared with established modalities [2], and emerging pilot studies suggest potential applications beyond diagnosis, such as monitoring tumor clearance after local therapies [2,42].

Nevertheless, the majority of published work does not address how LC-OCT can be systematically integrated into a standardized perioperative workflow for margin assessment. In contrast to algorithm- or performance-centered approaches, the present study focuses on workflow standardization and clinical implementation, integrating wide-field dermoscopic co-localization, structured acquisition paths, and AI-supported real-time visualization of suspicious margin areas. This shift from isolated diagnostic performance toward reproducible clinical integration constitutes the central novel contribution of the proposed “BCC-One-Stop-Shop” approach.

From a technical and organizational perspective, standardized in vivo LC-OCT–based margin assessment has the potential to reduce overall costs and resource utilization. By enabling more accurate preoperative delineation of tumor margins, unnecessary tissue removal may be avoided, which is particularly relevant in cosmetically and functionally sensitive areas. In addition, improved margin control prior to surgery may reduce the number of surgical stages, especially in micrographic surgery, thereby decreasing operating room time, histopathological workload, and personnel requirements. The digital nature of the workflow, including dermoscopic co-localization and AI-supported visualization, further supports efficient documentation and reproducibility, facilitating implementation in routine clinical settings without substantial additional infrastructure.

The ultimate goal should be complete BCC excision in a single surgical step—removing as much tissue as necessary, but as little as possible. Within this context, ex vivo LC-OCT imaging may offer additional confirmation of margin status. A particularly interesting development would also be the ability to confirm clearance at the deep margin of the ex vivo excised specimen and if needed, to enable immediate re-excision of residual tumor. However, several practical limitations of ex vivo LC-OCT imaging must be considered. Imaging freshly excised specimens through a flat glass window can be challenging due to uneven tissue surfaces, tissue folding, or trapped air bubbles at the tissue–glass interface, which may impair optical coupling and obscure visualization of the deep margin. These factors represent inherent technical constraints of ex vivo imaging and should be taken into account when interpreting deep margin findings.

We are currently conducting a large multicenter study to further evaluate the diagnostic accuracy, efficacy and safety of the “BCC-One-Stop-Shop-Method” and to directly compare it with the current gold standard, MMS. This effort aims to define the most effective strategies for integrating noninvasive dermatological imaging into surgical tumor treatment.

## Figures and Tables

**Table 1:** Key imaging parameters of the different types of images of the deepLive^TM^ video-dermoscope and the deepLive^TM^ LC-OCT device.

Imaging Parameters	LC-OCT	Color Surface Imaging (Secondary Path of the LC-OCT Probe)	Video-Dermoscopy
Resolution (µm)	1.3 × 1.1 (lateral × axial)	6	3.5
Numerical aperture	0.5	0.07	0.12
Field of view (mm)	1.2 × 0.4 (vertical) 1.2 × 0.5 (horizontal) 1.2 × 0.5 × 0.5 (3D)	2.6 (diameter)	13.3 × 8.9 (without mosaic)
Image dimensions (pixels)	2048 × 680 (vertical 2048 × 850 (horizontal) 1200 × 500 × 500 (3D)	400 × 400	5536 × 3692 (without mosaic)
Frame rate (frames per second)	8 (vertical and horizontal) 20 (3D)	8	8

## Data Availability

The data supporting the conclusions of this article will be made available by the authors upon reasonable request.

## Abbreviations

The following abbreviations are used in this manuscript:

AI	Artificial intelligence
BCC	Basal cell carcinoma
EADO	European Association of Dermato-Oncology
FOV	Field of view
LC-OCT	Line field confocal optical coherence tomography
NA	Numerical aperture
OCT	Optical coherence tomography
RANSAC	Random Sample Consensus
RCM	Reflectance confocal microscopy
SIFT	Scale-Invariant Feature Transform
SOP	Standard of procedure
